# The Roles of Blood Lipid-Metabolism Genes in Immune Infiltration Could Promote the Development of IDD

**DOI:** 10.3389/fcell.2022.844395

**Published:** 2022-02-09

**Authors:** Weihang Li, Ziyi Ding, Huan Zhang, Quan Shi, Dong Wang, Shilei Zhang, Songjie Xu, Bo Gao, Ming Yan

**Affiliations:** ^1^ Department of Orthopedic Surgery, Xijing Hospital, Air Force Medical University, Xi’an, China; ^2^ Department of Orthopaedics, Affiliated Hospital of Yanan University, Yanan, China; ^3^ Beijing Luhe Hospital, Capital Medical University, Beijing, China

**Keywords:** intervertebral disc degeneration, WGCNA, immune infiltration, diagnostic model, lipid-metabolism genes

## Abstract

**Objectives:** Intervertebral disc degeneration is a progressive and chronic disease, usually manifesting as low back pain. This study aimed to screen effective biomarkers for medical practice as well as figuring out immune infiltration situations between circulation and intervertebral discs.

**Methods:** Gene expression profiles of GSE124272 was included for differentially analysis, WGCNA and immune infiltration analysis from GEO database, and other GSE series were used as validation datasets. A series of validation methods were conducted to verify the robustness of hub genes, such as principal component analysis, machine learning models, and expression verification. Lastly, nomogram was established for medical practice.

**Results:** 10 genes were commonly screened *via* combination of DEGs, WGCNA analysis and lipid metabolism related genes. Furthermore, 3 hub gens CYP27A1, FAR2, CYP1B1 were chosen for subsequent analysis based on validation of different methods. GSEA analysis discovered that neutrophil extracellular traps formation and NOD-like receptor signaling pathway was activated during IDD. Immune infiltration analysis demonstrated that the imbalance of neutrophils and γδT cells were significantly correlated with IDD progression. Nomogram was established based on CYP27A1, FAR2, CYP1B1 and age, the calibration plot confirmed the stability of our model.

**Conclusion:** CYP27A1, FAR2, CYP1B1 were considered as hub lipid metabolism related genes (LMRGs) in the development of IDD, which were regarded as candidate diagnostic biomarkers especially in circulation. The effects are worth expected in the early diagnosis of IDD through detecting these genes in blood.

## Introduction

Low back pain, a worldwide and common medical problem in adult population, has been estimated that more than 80% of adults experienced at least once during their lifetime (Rubin, 2007). There are many causes of low back pain, while intervertebral disc degeneration, namely IDD, is considered as the most common trigger (Rigal et al., 2017). In clinic, IDD is a progressive and chronic disorder, together with alterations in muscle and skeleton, the progression may further result in herniation, spinal canal stenosis, and degenerative spondylolisthesis (Jin et al., 2013). Thus, the present management of IDD patients constitute a major socioeconomic issue. Currently, the main clinical therapeutic approaches regarding IDD include surgery and conservative treatment. The impacts of the alterations in biomechanics and long-term sequelae may be significant with surgical intervention, and conservative treatment may not ameliorate the symptoms immediately (Hermantin et al., 1999; Yorimitsu et al., 2001).

Accumulating evidences reported the etiology of IDD, such as loading changes, aging, smoking, poor nutrients supply, and hereditary aspects, among which the genetic factor is considered as essential risk factor accounting for more than 70% (Battié et al., 1995; Le Maitre et al., 2007; Kepler et al., 2013). Besides, As the largest avascular organ in body, Intervertebral disc (IVD) is consist of the central nucleus pulposus (NP), peripheral annulus fibrosus (AF) surrounding NP, together with the upper and lower cartilage endplate (EP) (Le Maitre et al., 2007), it is regarded as immune-privilege organ due to its special structure (Sun et al., 2020). Emerging studies have indicated immune infiltration had pivotal roles in the occurrence and progression of IDD, IDD had displayed distinct patterns of macrophages infiltration in IVD tissue (Ikeda et al., 1996; Koike et al., 2003); different cytokines secreted by immune cells were found participating IDD initiation and progression by regulating inflammatory response (Risbud and Shapiro, 2014). Most researches mainly focused on the study of immune cells within IVD tissue, while the expression situations of immune cells among blood, and the detailed immune infiltration correlations between blood and IVD tissues had hardly been reported. Therefore, more researches about the real biological significance behind genes in human blood of IDD need to be conducted and further make an understanding between blood and IVD tissue.

Lipid metabolism related genes, namely LMRGs, as the second messenger of intracellular signal transduction, lipids behave essential roles in different organelles (Efeyan et al., 2015). Researches about LMRGs were widely conducted in various diseases like degenerative alterations, atherosclerosis, diabetes, and different kinds of cancers (Santos and Schulze, 2012; Johnson and Stolzing, 2019; Chow et al., 2020; Thongnak et al., 2020). Recent years, LMRGs reprogramming has also been regarded as a novel hallmark of tumor malignancy, and the pivotal roles of LMRGs in the initiation, progression, and treatment of tumors have been proved (Cheng et al., 2018). Up to now, existed studies mainly focused on analysis of DEGs in IVD tissue, while the roles of blood LMRGs in the development of IDD has remained poorly understood. Besides, existed research had made great progress in diagnosis of disease through blood, the applications of predictive models have been verified in lung cancer through detecting hub genes from blood tissues (Schult et al., 2021), thus the blood tissue predictive model exhibit broad prospects in early prediction and diagnosis of diseases.

In this study, we combined differentially expressed analysis and weighted gene co-expression network analysis (WGCNA) from blood tissues of IDD patients, together with LMRGs to screen out hub LMRGs responsible for IDD progression, and make a comprehensive analysis of hub LMRGs by functional and enrichment analysis and immune infiltration analysis. Then a series of validation methods were performed to evaluate the reliability of these hub LMRGs, including principal component analysis, machine learning model, expression validation in separate dataset, and correlation analysis between clinical trait and LMRGs. Finally, this study constructed a nomogram model to predict the development of IDD, which may ultimately provide new ideas for early diagnosis of IDD through detecting the hub LMRGs in blood.

## Materials and Methods

### Microarray Datasets Acquisition

Microarray datasets were screened and downloaded from gene expression omnibus (GEO, https://www.ncbi.nlm.nih.gov/geo/), a functional public genomics database including high throughput sequencing data, chips as well as microarray data. The GSE124272 series was firstly extracted from GEO for DEGs and WGCNA analysis, which contained chips data of 16 whole bloods samples, among which 8 patients with IDD and 8 patients with non-degenerative IVD. GSE23130, GSE70362, GSE34095 and GSE147383 series were also obtained from GEO database, these all contained non-degenerative disc and degenerative IVD tissues with different pfirrmann grades, which were used for validation in this study, and to avoid clinical features bias among different researches.

### Gene Expression Profiles Preprocessing

This study downloaded two types of file format for analysis: “raw” data (“.CEL” file format) and “series_matrix_file” data (“.txt” file format) data, among which “raw” data included GSE23130, GSE34095, GSE70362, GSE147383, while “series_matrix_file” data contained GSE124272. The raw data were preprocessed with background correction and normalization using “RMA” algorithm (“affy”, “affyPLM” packages and “rma” function and in R), and then conducted quality control by normalized unscaled standard error (NUSE). Both two file formats were applied to convert probe sets into gene symbols according to manufacture-provided annotation files, probe sets without corresponding gene symbols were removed, and median expression value were retained for different probe sets targeting the same gene.

### Differentially Expressed Genes Identification

According to the pfirrman grade of criteria, grade I-II were regarded as non-degenerative IVD while grade III-V were considered as degenerative IVD. This study divided the patients into two groups based on this classification and then the differentially expressed genes (DEGs) between IDD and non-degenerative IVD patients from whole blood were screened through R (“limma” package). An adjusted *p*-value < 0.05 and |FC| (fold change) > 1.5 were set as cut-off threshold for statistically significant.

### Functional and Pathway Enrichment Analysis

DAVID database (database for annotation, visualization, and integrated discovery, https://david.ncifcrf.gov/) and R (“clusterProfiler” package) were both applied for DEGs, which were uploaded into DAVID and R environment to get functional annotations and interpretations, *p* < 0.05 was set as cut-off value. For gene set enrichment analysis (GSEA, http://software.broadinstitute.org/gsea/index.jsp), it was conducted to get more essential biological functions or pathways which may be ignored by differential analysis, based on all genes and phenotype. The annotated gene set c2.cp.kegg.v7.4 was chosen as reference information. *p* < 0.05, gene size > 20 and |enrichment scores| (ES) > 0.4 were considered as statistically significant.

### Evaluation of Immune Cells Profiles Among Blood Samples

Totally 22 types of immune infiltrating cells were assessed from blood tissues among GSE124272 (Wang et al., 2019), to get a comprehensive information about immune cells expression situation. In GSE124272, left medial cubital vein of participants were collected for further analysis. “Cibersort.R” code and standard immune cell expression file “LM22.txt” were obtained from official website (https://cibersort.stanford.edu/) and they were conducted by R. Then the abundance of immune cell members from mixed cells population were evaluated through gene expression profiles, and correlation heatmap, proportion situation as well as different expressions between immune cells and samples were fully analyzed and visualized.

### Weight Gene Co-Expression Network Analysis

All genes of whole blood from GSE124272 were included for WGCNA (“WGCNA” and related packages in R) analysis, to avoid the inaccurate results that may generate from the top of 5,000 genes (according to variance). Hierarchical clustering analysis was firstly performed to eliminate outliers from samples. Then soft threshold power was calculated and the optimal value was chosen for subsequent network construction, to get access to the real biological network state (scale-free network). The best appropriate soft threshold power was selected when scale-free index (*R* ^2^) reached 0.90 and mean connectivity approximate 0.

Weighted gene co-expression network was constructed through the optimal soft threshold power based on the correlations of gene expression, and co-expression modules were identified and clustered according to the similarity of each other. The minimum number of genes in each clustered module were set as 30. Then eigengenes adjacency were calculated by PCA dimension and correlated with each other, module-trait Pearson’s correlations were calculated to recognize relationships between modules and clinical traits, then hierarchical clustering analysis was also performed to display module eigengene and phenotype.

After identifying the relationships and correlations among these clustered modules and phenotype, gene significance (GS) and module membership (MM) between each module and IDD were calculated and visualized. Finally, the module with the highest correlation coefficient was selected as the key module and applied for further analysis.

### Hub Genes Signature Screening Among Lipid Metabolism Related Genes

This study obtained totally 750 lipid metabolism related genes (LMRGs) from the Reactome and KEGG repository. After identification of DEGs, and key module from WGCNA, this study aimed to analyze the relationships between LMRGs and IDD progression, especially in blood tissues. The hub genes were finally identified by taking the intersection of three parts of genes to obtain a more accurate and convincing result, based on Venn plot analysis (“VennDiagram” package in R).

### Establishment of Machine Learning to Verify the Reliability of Hub Genes

To verify the robust of these identified hub genes, they were applied to train machine learning model, including lasso regression, K-nearest neighbor algorithm, elasticnet regression, ridge regression, random forest algorithm, logistic regression, as well as support vector machine model (“caret”, “e1071”, “kernlab” packages in R). Five-fold cross validation was conducted in the training model to get the most suitable equation and the most accurate predicting results. The dot plot of each machine learning methods was displayed to assess the robustness of these hub genes.

### Hub Genes Validation and Principal Component Analysis Among Different Databases

Principal component analysis (PCA) was performed to reduce dimension of these hub genes, to observe whether they could distinguish the IDD patients from normal patients, “prcomp” and “princomp” functions in R were applied. The results were displayed in 3D scatter plot (“scatterplot3d” package in R). Then the hub genes expressions situation was evaluated by other GSE series GSE70362 to get more precise genes in IVD tissue.

### Clinical Traits and Hub Genes Correlation Analysis and Immune Infiltrating Analysis

The genes which were validated to have statistical significance in former step were then conducted to analyze the relationships with clinical traits, the GSE series contained age and gender information were chosen for analysis: GSE34095, GSE147383. Then the most correlated gene were further performed for immune infiltration analysis only in IDD patients, and IDD patients were divided into two groups according to the median value of the gene expression.

### Clinical Diagnostic Applications Based on Hub Lipid Metabolism Related Genes

Based on the hub LMRGs identified in this study, we calculated the predictive ability of each variable, ROC curve was plotted and area under curve (AUC) was calculated to evaluate the prediction results. Then we established an innovative nomogram, expected to make accurate diagnosis in early stage of IDD.

### Statistical Analysis

The data statistical analysis and visualization were conducted by R (version 4.1.2, based on different packages which mentioned above) and GraphPad Prism (version 8.0.1). The association between continuous variables was evaluated by Spearman’s correlation coefficient. One-way ANOVA analysis was selected flexibly when three or more groups to compare, and *t* test was used for statistical analysis between two groups, *p* < 0.05 was considered as statistically significant difference.

## Results

### Identification of DEGs and Enrichment Analysis in Intervertebral Disc Degeneration Patients

**FIGURE 1:**
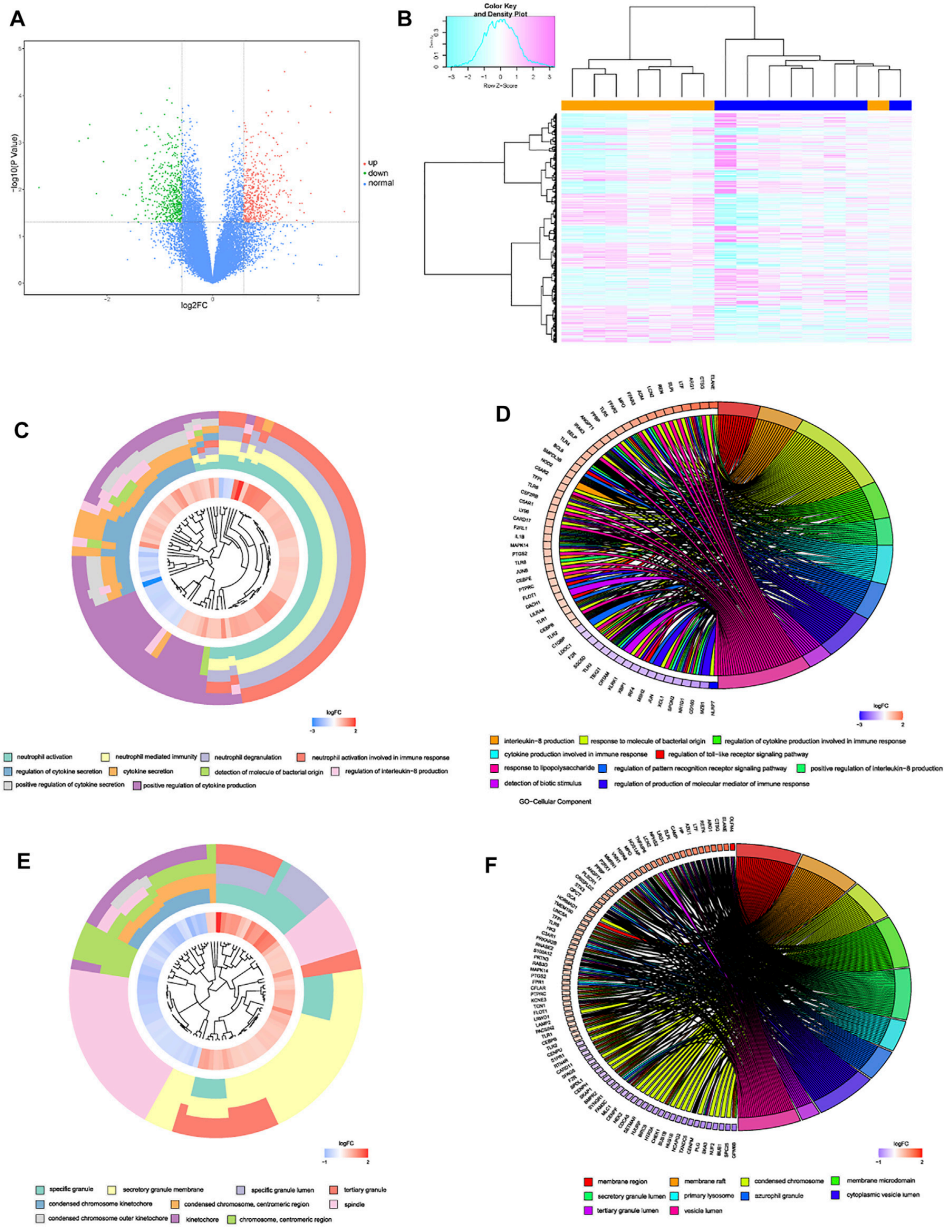
**(A)** volcano plot of DEGs, red dots represented up-regulated genes, green dots represented down-regulated genes, and black dots represented normal genes. **(B)** heatmap of DEGs in GSE124272. **(C–F)** enrichment cluster plot and chord plot of BPs and MFs from GO analysis, the first 10 terms were illustrated as enrichment cluster plot, and the next 10 terms were illustrated as chord plot.

**FIGURE 2:**
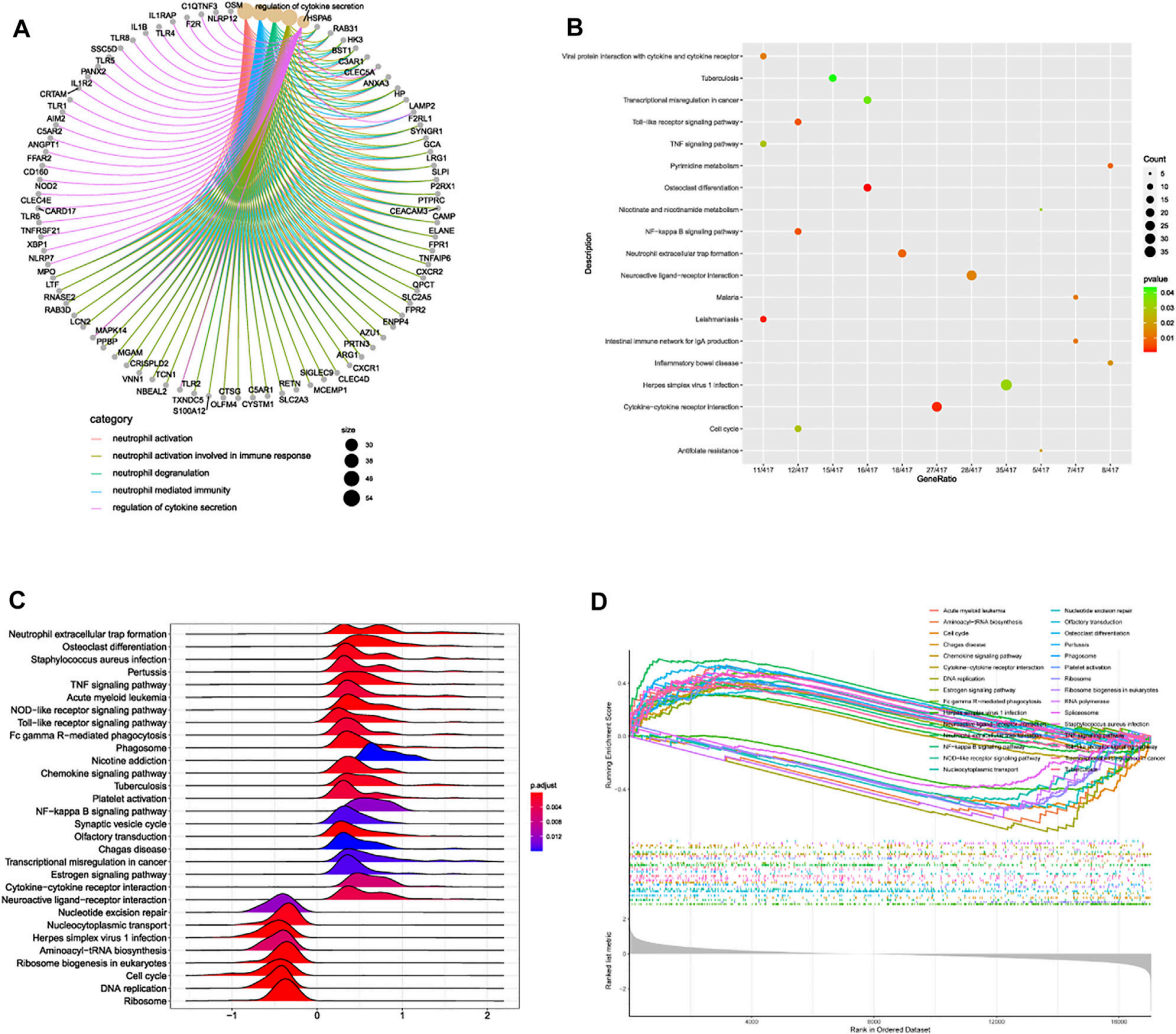
**(A)** association network diagram depicting the most correlated functions and corresponding genes. **(B)** bubble plot of pathway enrichment analysis. **(C)** ridge plot and **(D)** line graph of GSEA analysis.

After linear model fitting and bayes detection about gene expressions from two groups (IDD and normal group), and based on the cutoff criteria adjusted *p*-value < 0.05 and |FC| > 1.5, totally 1,053 genes were identified as DEGs, among which 509 up-regulated and 544 genes down-regulated genes. Volcano plot was plotted to exhibit gene distribution (Figure 1A), and hierarchical clustering analysis was conducted to verify the distinguishment ability of DEGs between IDD and normal patients (Figure 1B). The mutual up-regulated and down-regulated DEGs were then uploaded into DAVID database and R, to gain further insight and information about the potential functions of genes in the progression of IDD. GO terms included biological process (BP), cellular component (CC) and molecular function (MF), the first 10 functions of BP and CC were illustrated as enrichment cluster plot, and the next 10 functions were displayed as chord plot, as shown in Figures 1C–F. Results suggested that these DEGs were mainly involved in 127 BP terms (including neutrophil activation, regulation of cytokine secretion, regulation of interleukin-8 production, leukocyte activation involved in inflammatory response, and regulation of toll-like receptor signaling pathway, etc.), 26 CC terms (such as specific granule, spindle, membrane region, primary lysosome, and chromosomal region, etc.) and 1 MF term (immune receptor activity). Besides, based on adjusted *p*-value, functions mainly contained neutrophil activation, neutrophil degranulation, neutrophil mediated immunity, and regulation of cytokine secretion, related genes involved in these functions were also displayed, as shown in Figure 2A. KEGG results suggested that some signaling pathways were aberrantly changed in the progression of IDD, including osteoclast differentiation, cytokine-cytokine receptor interactions, NF-κB signaling pathway, toll-like receptor signaling pathway, pyrimidine metabolism, TNF signaling pathway, ferroptosis, etc (Figure 2B). As for GSEA, whole gene expressions were all included in analysis, the results were shown in Figures 2C,D, which suggested that nucleotide excision repair, nucleocytoplasmic transport, ribosome biogenesis in eukaryotes, etc. were enriched in normal group, while neutrophil extracellular traps formation, TNF signaling pathway, toll-like receptor signaling pathway, NOD-like receptor signaling pathway, NF-κB signaling pathway, and chemokine signaling pathway, etc. were enriched in IDD patients. Taken together, these results identified potential biomarkers and exhibited abnormal functions as well as signaling pathways involved in the progression of IDD.

### Immune Landscape Associated With Venous Blood From Intervertebral Disc Degeneration Patients

**FIGURE 3:**
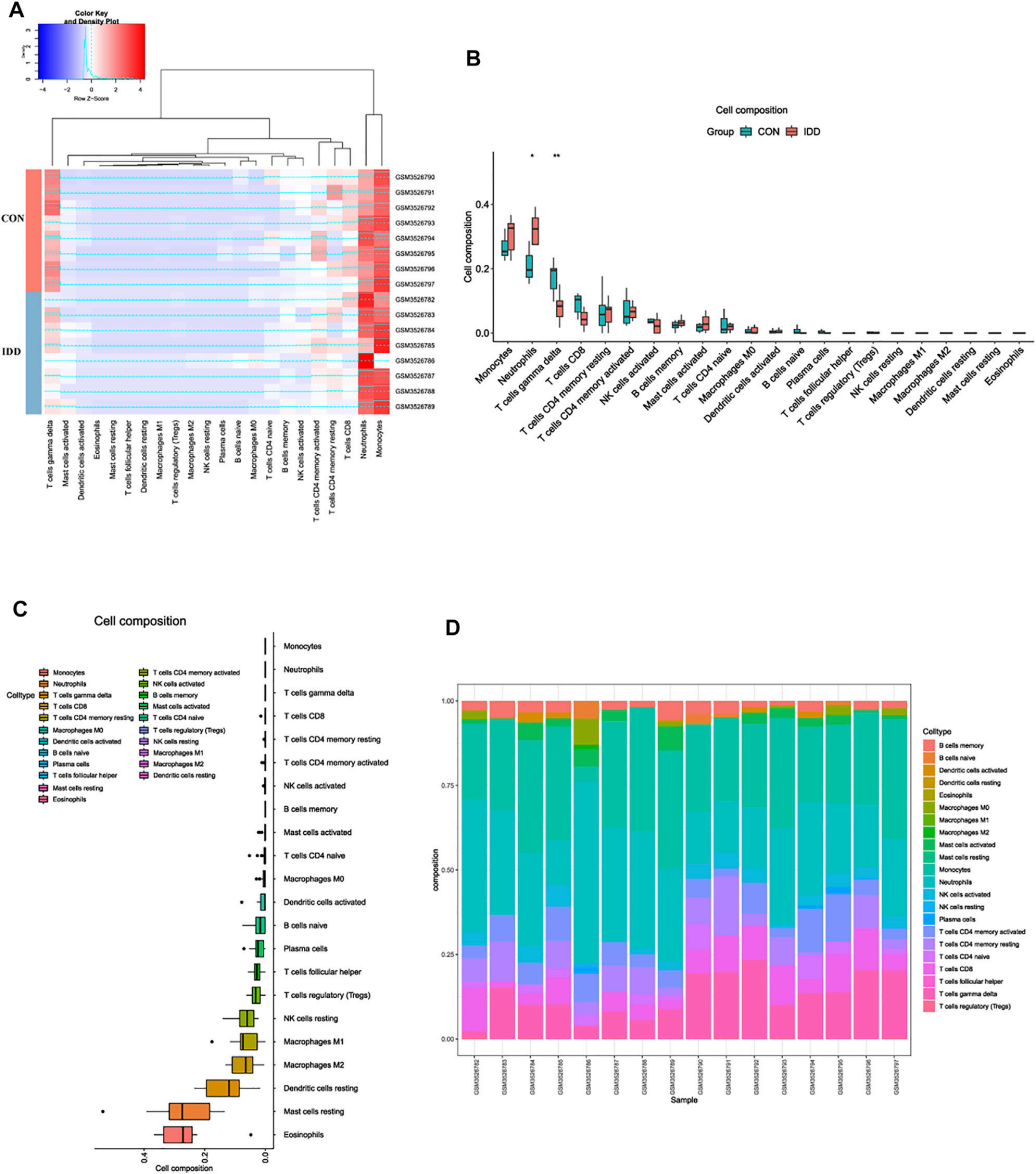
Analysis of immune landscape associated with IDD in blood tissue. **(A)** heatmap displaying the distribution of 22 types of immune cells in blood tissue of normal and IDD patients in GSE124272. **(B)** the relationship of immune infiltration levels between normal and IDD patients. **(C)** boxplot displaying the whole composition of immune cells in these patients. **(D)** pile-up histogram showing composition of immune cells in each sample.

Former enrichment analysis suggested that immune-related functions and signaling pathways were primarily enriched in IDD patients, compared to normal patients. To further discover the differential situations of immune landscape in venous blood tissues between IDD and normal patients, blood tissues microarray data from GSE124272 series was conducted for immune infiltration analysis. CIBERSORT method was performed *via* deconvolution algorithm, which was applied to estimate the relative proportion of 22 types of immune cells among two groups. The distribution of 22 types of immune cells in blood were clustered and shown in Figure 3A. The immune landscape results suggested that neutrophils were abnormally upregulated whereas T cells gamma delta (γδT cells) were downregulated in blood tissues of IDD patients (Figure 3B). Next, we analyzed the expression situation and proportion of immune cells in each sample, the detailed information about expressions of each immune cell were clearly visualized in Figures 3C,D, results illustrated that the composition of monocytes, neutrophils, γδT cells and CD8^+^ T cells were dominant in blood tissues, while NK cells resting, macrophages M2, dendritic cells resting, mast cells resting, and eosinophils were seldomly expressed in blood tissues.

### Construction of Weighted Gene Co-Expression Network

**FIGURE 4:**
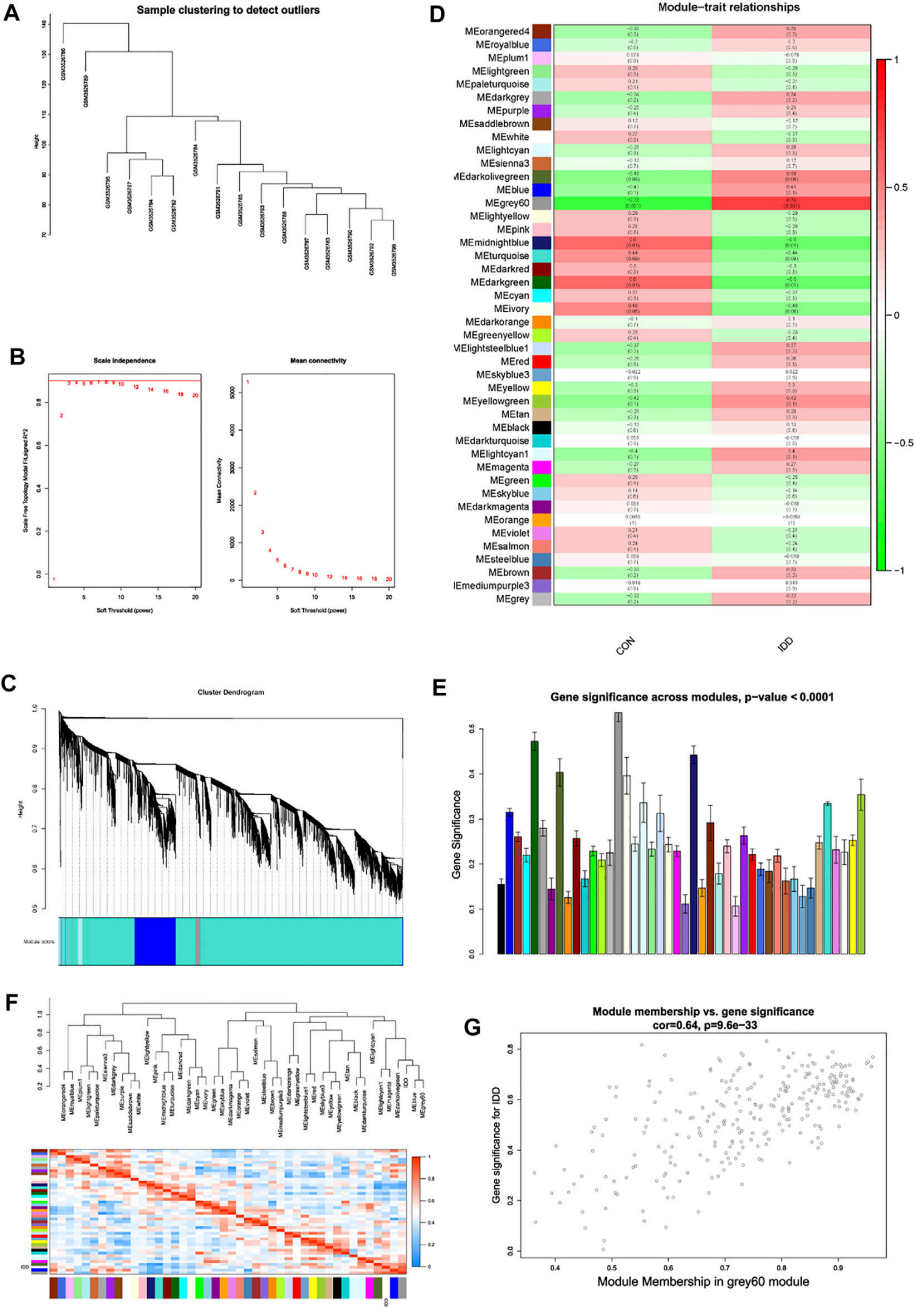
Weight gene co-expression network analysis. **(A)** sample clustering detection. **(B)** evaluation of soft threshold power, left panel represented scale-free fit index, and right panel represented mean connectivity of these values. **(C)** clustering dendrogram of all genes based on dissimilarity measure (1-TOM) and assignment modules, and highly interacted genes were clustered together. **(D)** module-trait relationships between different modules and feature. **(E)** histogram of gene significance across modules in IDD group. **(F)** cluster dendrogram and heatmap of adjacency in the eigengene network. **(G)** scatter plot showing correlations of gene significance for IDD vs. module membership in grey60 module.

Heterogeneity detection of each sample was conducted by hierarchical clustering analysis to check and remove outliers (Figure 4A), and all samples were included. Gene expression matrix of blood tissues which totally contained 18,672 genes with 16 samples were applied for WGCNA analysis. Soft threshold power was determined as 8 when *R* ^2^ reached 0.90 and mean connectivity was infinitely approximating 0 (Figure 4B). As a result, power value 8 was selected as the optimal value and pooled for WGCNA network construction.

Based on gene mutual co-expression situation, we then conducted hierarchical clustering tree analysis, to cluster genes and generate modules which interacted with each other. All genes were concluded into distinct co-expression modules, and totally 44 modules were identified, as shown in Figure 4C. Consequently, this study further identified interested modules in each subgroup.

### Key Module Identification of Intervertebral Disc Degeneration

After generation of WGCNA network data, module-trait relationships heatmap was then evaluated, to visualize the most correlated modules with each subgroup. As shown in Figure 4D, in the column of IDD group, results visualized that the grey60 (*p* = 0.001), midnightblue (*p* = 0.01), and darkgreen (*p* = 0.01) modules had strong correlations with IDD progression, genes in these modules could promote or suppress the development of IDD. Thereinto, grey60 module had the strongest correlation (r = 0.73) and the lowest *p* value (*p* = 0.001). Gene significance histogram (Figure 4E) of each module also proved the reliability of our results, and module eigengene adjacency was subsequently calculated to cluster the identified modules with IDD feature, and heatmap was plotted to visualize the interactions between different modules and IDD subgroup. As shown in Figure 4F, results implied that IDD group clustered with blue and grey60 module, suggesting these two eigenvalues had highly correlations with each other.

**FIGURE 5:**
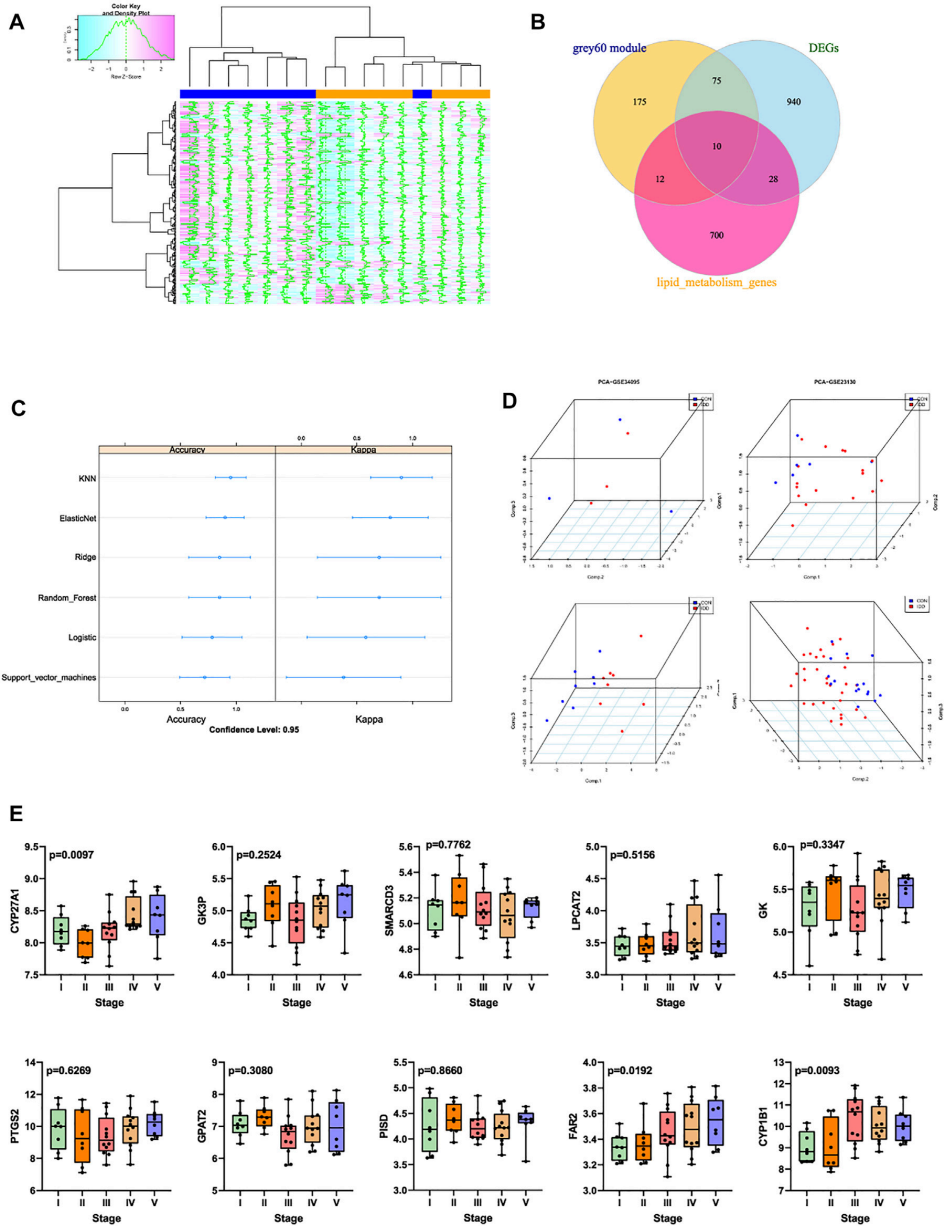
**(A)** grey60 module genes expression heatmap among IDD and normal groups. **(B)** Venn diagram indicating 10 genes were commonly expressed in three parts. **(C)** summary dot plot of different machine learning models. **(D)** 3D scatter plot after PCA dimension reduction of these 10 genes in different datasets. **(E)** expression validation of these 10 genes in GSE70362.

Then we performed module membership (MM) versus gene significance (GS) scatter plot (MM-GS plot) of grey60 module, results showed that MM was highly correlated with GS in grey60 module (Cor = 0.64, *p* = 9.6e-33) (Figure 4G). Heatmap results also validated the results, the color depth (red to blue) represented the strength of interactions among different eigenvalues, and IDD group was highly interacted with grey60 module. Based on gene expressions in grey60 module, we then performed hierarchical clustering analysis to figure out the separating capacity between IDD and normal group (Figure 5A). Results displayed that the genes in grey60 module could distinguish IDD group from normal group, which was consistent with above results. As a result, grey60 module was identified as key modules responsible for the progression of IDD.

### Selection and Validation of Lipid Metabolism Related Genes in Intervertebral Disc Degeneration Progression

Former results demonstrated that the expression levels of genes in grey60 module increased with the IDD progression, besides, 1,053 mutual DEGs were also screened to be highly related with IDD. Venn plot analysis was then performed by intersecting 1,053 DEGs, 272 genes in grey60 module, and 750 LMRGs obtained from Reactome and KEGG database. Totally 10 genes were commonly expressed in these 3 parts (Figure 5B), namely CYP27A1, GK3P, SMARCD3, LPCAT2, GK, PTGS2, GAPT2, PISD, FAR2, CYP1B1. Machine learning models were firstly established based on these 10 genes to verify the predictive ability of these genes, machine learning methods included lasso regression, K-nearest neighbor algorithm, elasticnet regression, ridge regression, random forest algorithm, logistic regression, as well as support vector machine model. After 5-fold cross validations, scatter plot of each model was displayed in Figure 5C.

Then the robust of these 10 genes were validated by PCA analysis among different databases, PCA was conducted to reduce dimension of these 10 genes into 3 principal components: PC1, PC2 and PC3, so that we could observe the spatial distribution and the clustering properties of data among IDD and normal groups. Based on gene expression values of matrix, results visualized that after dimension reduction of these 10 genes from GSE23130, GSE34095, GSE70362 and GSE124272, respectively, the 3 principal components PC1, PC2, PC3 could differentiate the normal samples from IDD samples clearly in three-dimensional coordinate system (Figure 5D).

Then the expression levels of these 10 genes were validated GSE70362, which contained gene expression situations in IVD tissues between normal (I-II pfirrman grade) and IDD (III-V pfirrman grade) patients. As shown in Figure 5E, the expression levels of CYP27A1 (*p* = 0.0097), FAR2 (*p* = 0.0192), CYP1B1 (*p* = 0.0093) were significantly upregulated in IDD patients than in normal patients. Thus CYP27A1, FAR2 and CYP1B1 were identified as hub genes, and applied for subsequent research.

### Correlation Analysis Between Hub Lipid Metabolism Related Genes and Clinical Traits

**FIGURE 6:**
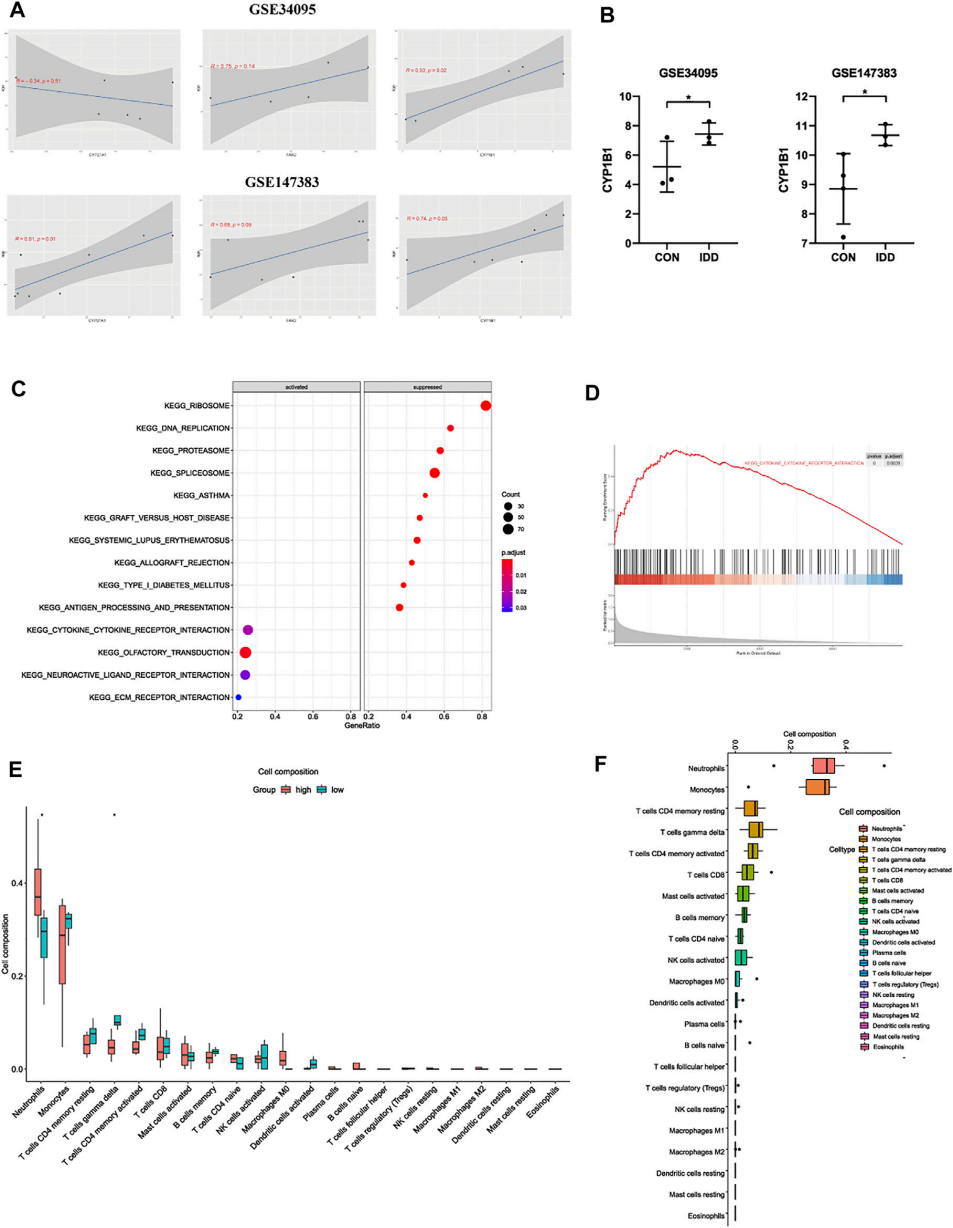
**(A)** the correlation analysis between CYP27A1, FAR2, CYP1B1 and clinical trait age in GSE34095 and GSE147383. **(B)** further expression validation of CYP1B1 in other datasets. **(C)** bubble plot and **(D)** line graph of GSEA analysis based on the expression of CYP1B1 in IDD patients. **(E)** the relationship of immune infiltration levels between CYP1B1-high and CYP1B1-low group in IDD patients. **(F)** boxplot displaying the whole composition of immune cells in IDD patients.

To verify the correlations between clinical traits and these 3 hub LMRGs, we chose GSE series which contained general information about patients including age and gender for analysis: GSE34095 and GSE147383. As shown in Figure 6A, after calculation of Pearson’s correlation coefficient and *p* value, results demonstrated that in GSE34095, GSE147383, the correlation between age and CYP27A1 were (Cor = −0.34, *p* = 0.51), and (Cor = 0.81, *p* = 0.01) respectively; the correlation between age and FAR2 were (Cor = 0.75, *p* = 0.14), and (Cor = 0.68, *p* = 0.09) respectively; and the correlation between age and CYP1B1 were (Cor = 0.93, *p* = 0.02), and (Cor = 0.74, *p* = 0.05) respectively. Among them, CYP1B1 was both positively correlated with age feature in these two series. Therefore, CYP1B1 was identified as the most correlated gene in the progression of IDD in this study, which possessed both characteristics that it was commonly expressed in different results, it was related with lipid metabolism, and it had positive correlation with clinical feature age.

### Functional Analysis and Immune Infiltration in Intervertebral Disc Degeneration Based on CYP1B1

After validations of three hub LMRGs, CYP1B1 was regarded as the most correlated LMRG among blood tissue in the progression of IDD. The expression situation was further confirmed in other GSE series to investigate the robustness of CYP1B1, and results demonstrated that CYP1B1 was significantly highly expressed both in GSE34095 and GSE147383 (Figure 6B). Subsequently, to elucidate the potential regulatory mechanisms of CYP1B1 among blood in the progression of IDD, microarray data of blood tissues GSE124272 was used for GSEA and immune infiltration analysis. IDD patients were divided into two groups according to the median expression levels of CYP1B1: high-CYP1B1 group and low-CYP1B1 group. As shown in Figures 6C,D, GSEA results indicated that “type I diabetes mellitus”, “DNA replication”, “graft versus host disease”, “herpes simplex virus I infection”, and “allograft rejection”, etc were enriched in CYP1B1-low group, while “neutrophil extracellular trap formation”, “osteoclast differentiation”, “Fc gamma R-mediated phagocytosis”, “transcriptional mis-regulation in cancer”, etc were enriched in CYP1B1-high group, which contained several immune-related pathways. Therefore, we next analyzed the relationships between immune infiltration and CYP1B1 expression. As shown in Figure 6E, immune results demonstrated that the infiltration levels of neutrophils were positively correlated with CYP1B1 expression, while γδT cells were negatively correlated with CYP1B1 expression. Besides, we further evaluated the expression situation and proportion of immune cells in CYP1B1-related groups from IDD patients, and results indicated that in blood tissues of IDD patients, neutrophils, monocytes, T cells CD4 memory resting, and γδT cells were dominant, while eosinophils, mast cells resting, dendritic cells resting were hardly existed (Figure 6F).

### Construction of Nomogram to Predict Intervertebral Disc Degeneration

**FIGURE 7:**
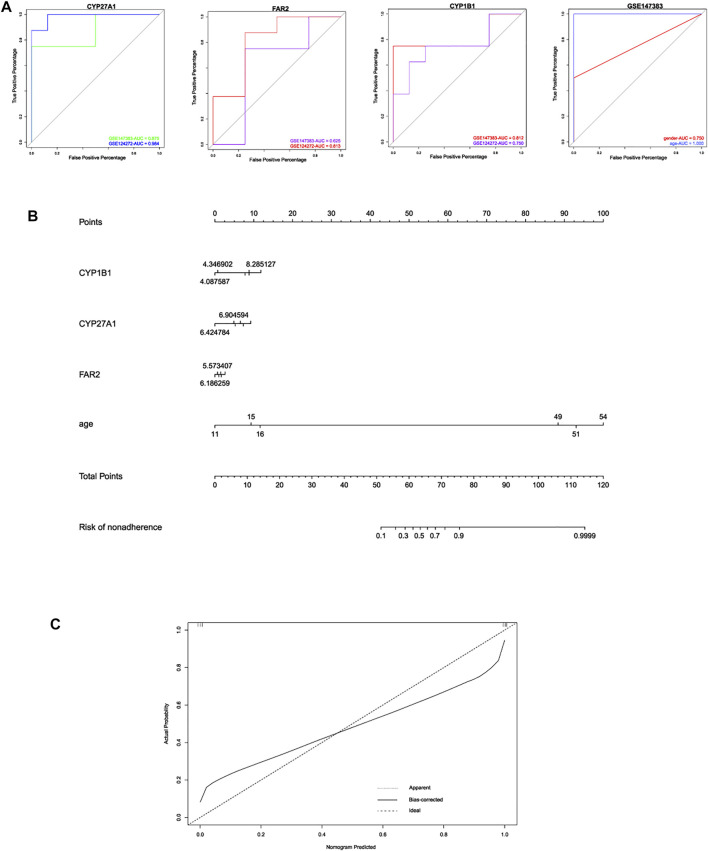
**(A)** ROC curve analysis of these 3 hub LMRGs for predicting IDD in different datasets. **(B)** construction of nomogram model based on 3 hub LMRGs and clinical trait age. **(C)** calibration plot of nomogram for evaluating robustness of the prediction.

Based on the three hub LMRGs identified in this study, we conducted ROC curve analysis to evaluate the predictive ability of each feature, including CYP27A1, FAR2, CYP1B1, gender and age. As shown in Figure 7A, results illustrated that AUC of CYP27A1 was 0.875, 0.984; AUC of FAR2 was 0.625, 0.813; AUC of CYP1B1 was 0.812, 0.750, respectively. Then we further combined the traditional clinical feature of age with the expression levels of hub LMRGs CYP27A1, FAR2, CYP1B1, to construct a nomogram model to predict IDD progression (Figures 7B,C), calibration plots were used to visualize the performance of the nomograms, and the results verified the ability of our model in terms of predicting IDD. Nomogram model depicted that higher age, higher expression of CYP27A1, FAR2, CYP1B1 in blood could result in higher probability of IDD initiation.

## Discussion

IDD is one of the main contributors to low back pain, and the cause of IDD is multifactorial with diversity of lead factors (Sun et al., 2013). Due to the lack of more detailed understanding of the underlying pathological mechanisms of IDD, the adverse effects of surgical intervention as well as conservative treatment all prompt researchers to figure out novel approaches in the treatment of IDD, as well as the early diagnostic methods, among which the genetic factor is the most essential aspect (Battié et al., 1995; Le Maitre et al., 2007; Kepler et al., 2013). WGCNA is a powerful holistic research tool for data mining, the mathematical theory was firstly proposed in 2005 (Zhang and Horvath, 2005), and the applications of WGCNA together with microarray technology have been witnessed widely in recent years in various tumor or non-tumor diseases, including primary myelofibrosis, osteosarcoma, gastric cancer, chronic obstructive pulmonary disease, etc. (Li et al., 2021a; Li et al., 2021b; Chen et al., 2021; Li et al., 2021c; Deng et al., 2021). In a nutshell, WGCNA builds a bridge between sample characteristics and gene expression profiles, which was performed in this study, together with other analytical methods. To our knowledge, the application of WGCNA in the identification of IDD have hardly been reported and fully analyzed, therefore, future biomarkers need comprehensive and insight analysis instead of simplistic differential analysis between IDD and normal patients.

Previous researches mainly focused on identification of hub genes from IVD tissue, whereas the biomarker of IDD in blood tissues and the relationships between IVD tissues and blood tissues were hardly analyzed. Besides, existed studies reported the importance of IVD as an immune privilege organ, which pointed the tight connections between immune infiltration and IVD tissue (Sun et al., 2020). However, the detailed information of immune situations in blood tissues of IDD patients remained unclear. As a result, this study focused on the alterations of gene functions among blood tissues of IDD patients, and combined analysis of DEGs and WGCNA to identify the potential biomarkers related to IDD in blood tissue.

In current study, a total of 1,053 DEGs were screened, GO analysis displayed that these DEGs were mainly involved in neutrophil activation, regulation of cytokine secretion, regulation of interleukin-8 production, and immune receptor activity, suggesting that the functions of DEGs primarily possessed immune-related functions, and these immune functions were highly correlated with IDD. KEGG results indicated that DEGs were aberrantly activated in several signaling pathways including osteoclast differentiation, cytokine-cytokine receptor interactions, NF-κB signaling pathway, toll-like receptor signaling pathway and TNF signaling pathway. Consequently, these results proved the connections between blood and IVD tissues, that alterations of immune functions as well as signaling pathways in blood tissues were highly correlated with IVD degeneration. And we considered that the aberrant alterations among blood tissues were early occurred than IVD degeneration, which may be regarded as the early caution in the development of IDD. As for GSEA, whole genes were involved and analyzed to figure out other essential signaling pathways which may be ignored by DEGs, and results indicated that in addition to signaling pathways which identified above, neutrophil extracellular traps formation and NOD-like receptor signaling pathway were also activated in IDD. NOD-like receptor was regarded as the essential receptor mediating immune recognition, together with Toll-like receptor. So, GSEA results further pointed the relationships between immune-related signaling pathways and IDD. In terms of WGCNA analysis, results revealed that 44 distinct clustered co-expression modules were generated, among which grey60 module had the highest correlation coefficients and the lowest *p* value, which was identified as the most correlated module in the progression of IDD. The module-trait and module eigengene adjacency heatmap confirmed our results. Besides, hierarchical clustering analysis of the genes in gery60 module also visualized that the gene expression levels could significantly distinguish IDD group from normal group.

After figuring that DEGs were tightly related with abnormal activation of immune functions in the development of IDD, this study then analyzed the differences of immune infiltrating cells in blood tissues between IDD and normal patients. Our results demonstrated the imbalance of neutrophils and γδT cells were significantly correlated with IDD progression. Neutrophils are the predominant leukocyte population in human blood, and are also absolutely pivotal part of the innate immune system (Castanheira and Kubes, 2019). Neutrophil could activate resident cells to generate inflammatory mediators like chemokines and cytokines, it could be regarded as one of the several inflammatory indicators used to determine systematic inflammatory responses (Sethi et al., 2012; Pittman and Kubes, 2013), which has been investigated in many diseases, such as autoimmune disease, fibromyalgia, infections, various malignancies, chronic obstructive pulmonary disease, etc. Besides, it behaved wide range of effect mechanisms against pathogens, including phagocytosis, and the production of reactive oxygen species, proteases, and neutrophil extracellular traps formation (Nathan, 2006). Our findings that neutrophils were up-regulated in IDD patients were consistent with previous research: a cross-sectional study about neutrophil/lymphocyte ratio (NLR) by Bozkurt, et al. reported a significant correlation between NLR and pre- and post-operative VAS scores of IDD patients under surgery (Bozkurt et al., 2019). As the largest avascular organ, IVD is considered as immune-privilege organ due to its special structure, accumulating evidences suggested the mechanism which limited immunocytes and immune mediators entering NP tissue in IVD could be defined as blood-NP barrier, a complex composition of physical and molecular factors (Sun et al., 2020), while blood vessels were crucial channel of immunocytes infiltration (Johnson et al., 2005). Studies also indicated that when tear of annulus fibrosus (AF) caused by physical factors or extracellular matrix degradation, the fissure of AF was chemically and mechanically conducive to the ingrowth of blood vessels (Yasuma et al., 1986; Vernon-Roberts et al., 2007; Stefanakis et al., 2012). Synthesizing above literature evidences and our results, we deduced that neutrophils could arrive and accumulate at degenerative IVD tissue through blood vessels, and directly acted on IVD by releasing related inflammatory factors, thereby forming a vicious cycle, and further damage the immune-privilege of IVD. This finding pointed out the importance of neutrophil infiltration in mediating the development of IDD. In addition, results displayed that γδT cells were down-regulated in IDD patients, γδT cells were unconventional subset of T lymphocytes which behave pivotal roles in homeostasis of the immune system (Borger et al., 2019; Qi et al., 2021). The applications of γδT cells were regarded as the key to immune cell therapy for different neoplasms, such as melanoma, gastric cancer, and colorectal cancer (Donia et al., 2012; Meraviglia et al., 2017; Wang et al., 2017). While the roles of γδT cells on tumors may have opposite effects due to different γδT cell subsets (Sebestyen et al., 2020). To the best knowledge, the roles of γδT cells in the development of IDD has not been reported before, since immune infiltrations existed in IVD and our finding that γδT cells were down-regulated in IDD, more researches about the interactions need to be conducted between γδT cell subsets and IDD.

3 LMRGs, namely CYP27A1, FAR2, and CYP1B1 were significantly highly expressed in both in blood and IVD tissue, which were considered as hub LMRGs for IDD. Several studies have reported the essential effects between lipid metabolisms and immune infiltration (Ringel et al., 2020; Wang et al., 2020); besides, lipid metabolisms factors were also reported to be highly correlated with IDD, such as weight, BMI, and serum lipid levels (Zhang et al., 2019). All these proved the importance of lipids metabolisms in IDD. This study also provided evidence of correlations between 3 hub LMRGs and IDD. We then verified the correlations between clinical traits and 3 LMRGs, results revealed that CYP1B1 was both highly correlated with age in different GSE series. Thus, CYP1B1 was considered as the most significant gene in the development of IDD. Functional and immune infiltration analysis in IDD patients were further conducted based on the expression levels of CYP1B1. Results demonstrated that CYP1B1-high group also mediated several immune reactions like neutrophil extracellular traps formation and Fc gamma R-mediated phagocytosis. Immune infiltration analysis displayed neutrophils infiltrations were significantly involved in CYP1B1-high group, which were consistent with our previous results, that IDD patients with CYP1B1-high group were liable to mediate immune response, and easier to develop cascade reactions and further promote IDD.

CYP1B1, one of the member of cytochrome P450 family proteins, located on chromosome 2p22-21 and encoded a protein of 543 ammino acids Unlike most members of CYP family, CYP1B1 was not expressed in human liver while expressed in many extrahepatic tissues, including lung, colon, kidney, etc. (D'Uva et al., 2018). Different studies had reported the essential roles of CYP1B1 in different diseases like metabolism diseases, hypertension, renal insufficiency, and cancers (D'Uva et al., 2018), thus, CYP1B1 was considered as potential effective biological target for the treatment of a variety diseases (Li et al., 2017). Retinas from CYP1B1-deficient mice showed decreased blood vessels density and inability to form new blood vessels; similarly, endothelial cells lacking CYP1B1 reduced the angiogenic activity *in vitro*, these studies indicated that CYP1B1 may induce angiogenesis (Tang et al., 2009). In terms of inflammation and immune infiltration, many natural CYP1B1 inhibitors possessed significant anti-inflammatory and anti-vascular properties, suggesting the pivotal roles of CYP1B1 in pathological inflammation and angiogenesis (Tang et al., 2010); macrophages from CYP1B1-deficient mice had impaired phagocytosis of apoptotic necrotic cells and opsonized cells, illustrating CYP1B1 behaved roles in macrophage activity (Ward et al., 2004). Based on the results in this study, we hypothesized that CYP1B1 may behave roles in stimulating immune infiltration and angiogenesis of IVD tissues and thus lead into progression of IDD. To the best knowledge, the correlations between CYP1B1 and IDD have not been reported yet, thus our findings in this study filled the gap between them and demonstrated the essential roles of CYP1B1 between immune infiltration and IDD. Furthermore, based on the extensive research about CYP1B1 inhibitors in different kinds of diseases, whether CYP1B1 inhibitor could also ameliorate the development of IDD is really a worth studying field.

To further explore the clinical application value of these 3 hub LMRGs, we calculated the predictive ability of single variables of CYP271, FAR2, CYP1B1, and ROC curve was plotted to reflect the relationship between sensitivity and specificity and judge the diagnostic value for each variable. AUC of each ROC curve (> 0.750) suggested a high predictive results of each single variable. Additionally, we combined the hub LMRGs together with clinical trait age, to establish a nomogram model, expecting to make accurate a diagnosis in early stage of IDD, through intaking age information and detecting 3 hub LMRGs in blood tissue. Consequently, the advanced age, high expression of CYP27A1, FAR2 and CYP1B1 in blood could be served as risk factor of IDD. Calibration plot confirmed the reliability of our nomogram model, which could be applied for clinical utility in the future.

Finally, although this study found the essential connections between LMRGs and IDD, and clinical trait age was included for analysis, we had to admit our limitations: e.g., the existed GEO series did not have enough samples, which may cause statistical error. Besides, the existed data did not contain enough general clinical information about patients, only few series had age information, which made it difficult to include different factors for analysis. Worthy mentioning that, our group have begun collecting IVD and blood tissues from patients, together with recording different general clinical information like age, weight, BMI, serum lipids, IDD grade, even their works and customary posture, aiming to fully analyze the predictive model through including genes expressions and different factors, so that constantly optimize our prediction model.

## Conclusion

Overall, 1,053 DEGs were analyzed by differentially analysis, 272 genes in grey60 module were identified by WGCNA analysis, finally, 10 genes were commonly expressed in DEGs, grey60 module as well as LMRGs. CYP27A1, FAR2 and CYP1B1 genes were further considered as hub LMRGs and diagnostic biomarkers in the progression of IDD, which were both highly expressed in other datasets, and were validated by PCA and machine learning model. Finally, a nomogram model was established based on the expression of these 3 hub LMRGs, together with clinical trait age, expecting to diagnose IDD in early stage through detecting these genes in blood.

## Data Availability

The datasets presented in this study can be found in online repositories. The names of the repository/repositories and accession number(s) can be found in the article/supplementary material.

## References

[B1] Battié M. C. Videman T. Gibbons L. E. Fisher L. D. Manninen H. Gill K. (1995). 1995 Volvo Award in Clinical Sciences. Determinants of Lumbar Disc Degeneration. A Study Relating Lifetime Exposures and Magnetic Resonance Imaging Findings in Identical Twins. Spine (Phila Pa 1976) 20 (24), 2601–2612. PubMed PMID: 8747238. 8747238

[B2] Borger J. G. Lau M. Hibbs M. L. (2019). The Influence of Innate Lymphoid Cells and Unconventional T Cells in Chronic Inflammatory Lung Disease. Front. Immunol. 10, 1597. PubMed PMID: 31354734. 10.3389/fimmu.2019.01597 31354734 PMC6637857

[B3] Bozkurt H. Arac D. Cigdem B. (2019). The Effect of the Preoperative Uric Acid Level and Neutrophil Lymphocyte Ratio on Preoperative and Postoperative Visual Pain Scores in Patients with Lumbar Disc Hernia: a Cross-Sectional Study. Turkish Neurosurg. 29 (5), 705–709. PubMed PMID: 30900735. 10.5137/1019-5149.JTN.25897-19.2 30900735

[B4] Castanheira F. V. S. Kubes P. (2019). Neutrophils and NETs in Modulating Acute and Chronic Inflammation. Blood 133 (20), 2178–2185. PubMed PMID: 30898862. 10.1182/blood-2018-11-844530 30898862

[B5] Chen H. Sun Q. Zhang C. She J. Cao S. Cao M. (2021). Identification and Validation of CYBB, CD86, and C3AR1 as the Key Genes Related to Macrophage Infiltration of Gastric Cancer. Front. Mol. Biosci. 8, 756085. PubMed PMID: 34950700. 10.3389/fmolb.2021.756085 34950700 PMC8688826

[B6] Cheng C. Geng F. Cheng X. Guo D. (2018). Lipid Metabolism Reprogramming and its Potential Targets in Cancer. Cancer Commun. 38 (1), 27. PubMed PMID: 29784041. 10.1186/s40880-018-0301-4 PMC5993136 29784041

[B7] Chow Y.-L. Teh L. K. Chyi L. H. Lim L. F. Yee C. C. Wei L. K. (2020). Lipid Metabolism Genes in Stroke Pathogenesis: The Atherosclerosis. Curr. Pharm. Des. 26 (34), 4261–4271. PubMed PMID: 32534558. 10.2174/1381612826666200614180958 32534558

[B8] D'Uva G. Baci D. Albini A. Noonan D. M. (2018). Cancer Chemoprevention Revisited: Cytochrome P450 Family 1B1 as a Target in the Tumor and the Microenvironment. Cancer Treat. Rev. 63, 1–18. PubMed PMID: 29197745. 10.1016/j.ctrv.2017.10.013 29197745

[B9] Deng M. Yin Y. Zhang Q. Zhou X. Hou G. (2021). Identification of Inflammation-Related Biomarker Lp-PLA2 for Patients with COPD by Comprehensive Analysis. Front. Immunol. 12, 670971. PubMed PMID: 34093570. 10.3389/fimmu.2021.670971 34093570 PMC8176901

[B10] Donia M. Ellebaek E. Andersen M. H. Straten P. t. Svane I. M. (2012). Analysis of Vδ1 T Cells in Clinical Grade Melanoma-Infiltrating Lymphocytes. Oncoimmunology 1 (8), 1297–1304. PubMed PMID: 23243593. 10.4161/onci.21659 23243593 PMC3518502

[B11] Efeyan A. Comb W. C. Sabatini D. M. (2015). Nutrient-sensing Mechanisms and Pathways. Nature 517 (7534), 302–310. PubMed PMID: 25592535. 10.1038/nature14190 25592535 PMC4313349

[B12] Hermantin F. U. Peters T. Quartararo L. Kambin P. (1999). A Prospective, Randomized Study Comparing the Results of Open Discectomy with Those of Video-Assisted Arthroscopic Microdiscectomy*†. J. Bone Jt. Surg. 81 (7), 958–965. PubMed PMID: 10428127. 10.2106/00004623-199907000-00008 10428127

[B13] Ikeda T. Nakamura T. Kikuchi T. Umeda S. Senda H. Takagi K. (1996). Pathomechanism of Spontaneous Regression of the Herniated Lumbar Disc. J. Spinal Disord. 9 (2), 136–140. PubMed PMID: 8793781. 10.1097/00002517-199604000-00009 8793781

[B14] Jin L. Shimmer A. L. Li X. (2013). The challenge and Advancement of Annulus Fibrosus Tissue Engineering. Eur. Spine J. 22 (5), 1090–1100. PubMed PMID: 23361531. 10.1007/s00586-013-2663-2 23361531 PMC3657039

[B15] Johnson A. A. Stolzing A. (2019). The Role of Lipid Metabolism in Aging, Lifespan Regulation, and Age-Related Disease. Aging Cell 18 (6), e13048. PubMed PMID: 31560163. 10.1111/acel.13048 31560163 PMC6826135

[B16] Johnson W. E. Caterson B. Eisenstein S. M. Roberts S. (2005). Human Intervertebral Disc Aggrecan Inhibits Endothelial Cell Adhesion and Cell Migration *In Vitro* . Spine (Phila Pa 1976) 30 (10), 1139–1147. PubMed PMID: 15897827. 10.1097/01.brs.0000162624.95262.73 15897827

[B17] Kepler C. K. Ponnappan R. K. Tannoury C. A. Risbud M. V. Anderson D. G. (2013). The Molecular Basis of Intervertebral Disc Degeneration. Spine J. 13 (3), 318–330. PubMed PMID: 23537454. 10.1016/j.spinee.2012.12.003 23537454

[B18] Koike Y. Uzuki M. Kokubun S. Sawai T. (2003). Angiogenesis and Inflammatory Cell Infiltration in Lumbar Disc Herniation. Spine 28 (17), 1928–1933. PubMed PMID: 12973136. 10.1097/01.brs.0000083324.65405.ae 12973136

[B19] Le Maitre C. L. Pockert A. Buttle D. J. Freemont A. J. Hoyland J. A. (2007). Matrix Synthesis and Degradation in Human Intervertebral Disc Degeneration. Biochem. Soc. Trans. 35 (Pt 4), 652–655. PubMed PMID: 17635113. 10.1042/BST0350652 17635113

[B20] Li F. Zhu W. Gonzalez F. J. (2017). Potential Role of CYP1B1 in the Development and Treatment of Metabolic Diseases. Pharmacol. Ther. 178, 18–30. PubMed PMID: 28322972. 10.1016/j.pharmthera.2017.03.007 28322972 PMC5600638

[B21] Li W. Zhao Y. Wang D. Ding Z. Li C. Wang B. (2021). Transcriptome Research Identifies Four Hub Genes Related to Primary Myelofibrosis: a Holistic Research by Weighted Gene Co-expression Network Analysis. Aging 13 (19), 23284–23307. PubMed PMID: 34633991. 10.18632/aging.203619 34633991 PMC8544335

[B22] Li W. Ding Z. Wang D. Li C. Pan Y. Zhao Y. (2021). Ten-gene Signature Reveals the Significance of Clinical Prognosis and Immuno-Correlation of Osteosarcoma and Study on Novel Skeleton Inhibitors Regarding MMP9. Cancer Cel Int 21 (1), 377. PubMed PMID: 34261456. 10.1186/s12935-021-02041-4 PMC8281696 34261456

[B23] Li W. Yuan B. Zhao Y. Lu T. Zhang S. Ding Z. (2021). Transcriptome Profiling Reveals Target in Primary Myelofibrosis Together with Structural Biology Study on Novel Natural Inhibitors Regarding JAK2. Aging 13 (6), 8248–8275. PubMed PMID: 33686952. 10.18632/aging.202635 33686952 PMC8034969

[B24] Meraviglia S. Lo Presti E. Tosolini M. La Mendola C. Orlando V. Todaro M. (2017). Distinctive Features of Tumor-Infiltrating γδ T Lymphocytes in Human Colorectal Cancer. Oncoimmunology 6 (10), e1347742. PubMed PMID: 29123962. 10.1080/2162402X.2017.1347742 29123962 PMC5665062

[B25] Nathan C. (2006). Neutrophils and Immunity: Challenges and Opportunities. Nat. Rev. Immunol. 6 (3), 173–182. PubMed PMID: 16498448. 10.1038/nri1785 16498448

[B26] Pittman K. Kubes P. (2013). Damage-associated Molecular Patterns Control Neutrophil Recruitment. J. Innate Immun. 5 (4), 315–323. PubMed PMID: 23486162. 10.1159/000347132 23486162 PMC6741494

[B27] Qi C. Wang Y. Li P. Zhao J. (2021). Gamma Delta T Cells and Their Pathogenic Role in Psoriasis. Front. Immunol. 12, 627139. PubMed PMID: 33732249. 10.3389/fimmu.2021.627139 33732249 PMC7959710

[B28] Rigal J. Léglise A. Barnetche T. Cogniet A. Aunoble S. Le Huec J. C. (2017). Meta-analysis of the Effects of Genetic Polymorphisms on Intervertebral Disc Degeneration. Eur. Spine J. 26 (8), 2045–2052. PubMed PMID: 28551829. 10.1007/s00586-017-5146-z 28551829

[B29] Ringel A. E. Drijvers J. M. Baker G. J. Catozzi A. García-Cañaveras J. C. Gassaway B. M. (2020). Obesity Shapes Metabolism in the Tumor Microenvironment to Suppress Anti-tumor Immunity. Cell 183 (7), 1848–1866. PubMed PMID: 33301708. 10.1016/j.cell.2020.11.009 33301708 PMC8064125

[B30] Risbud M. V. Shapiro I. M. (2014). Role of Cytokines in Intervertebral Disc Degeneration: Pain and Disc Content. Nat. Rev. Rheumatol. 10 (1), 44–56. PubMed PMID: 24166242. 10.1038/nrrheum.2013.160 24166242 PMC4151534

[B31] Rubin D. I. (2007). Epidemiology and Risk Factors for Spine Pain. Neurol. Clin. 25 (2), 353–371. PubMed PMID: 17445733. 10.1016/j.ncl.2007.01.004 17445733

[B32] Santos C. R. Schulze A. (2012). Lipid Metabolism in Cancer. FEBS J. 279 (15), 2610–2623. PubMed PMID: 22621751. 10.1111/j.1742-4658.2012.08644.x 22621751

[B33] Schult T. A. Lauer M. J. Berker Y. Cardoso M. R. Vandergrift L. A. Habbel P. (2021). Screening Human Lung Cancer with Predictive Models of Serum Magnetic Resonance Spectroscopy Metabolomics. Proc. Natl. Acad. Sci. USA 118 (51), e2110633118. PubMed PMID: 34903652. 10.1073/pnas.2110633118 34903652 PMC8713787

[B34] Sebestyen Z. Prinz I. Déchanet-Merville J. Silva-Santos B. Kuball J. (2020). Translating Gammadelta (γδ) T Cells and Their Receptors into Cancer Cell Therapies. Nat. Rev. Drug Discov. 19 (3), 169–184. PubMed PMID: 31492944. 10.1038/s41573-019-0038-z 31492944

[B35] Sethi S. Mahler D. A. Marcus P. Owen C. A. Yawn B. Rennard S. (2012). Inflammation in COPD: Implications for Management. Am. J. Med. 125 (12), 1162–1170. PubMed PMID: 23164484. 10.1016/j.amjmed.2012.06.024 23164484

[B36] Stefanakis M. Al-Abbasi M. Harding I. Pollintine P. Dolan P. Tarlton J. (2012). Annulus Fissures Are Mechanically and Chemically Conducive to the Ingrowth of Nerves and Blood Vessels. Spine (1976) 37 (22), 1883–1891. PubMed PMID: 22706090. 10.1097/BRS.0b013e318263ba59 22706090

[B37] Sun Z. Wan Z. Y. Guo Y. S. Wang H. Q. Luo Z. J. (2013). FasL on Human Nucleus Pulposus Cells Prevents Angiogenesis in the Disc by Inducing Fas-Mediated Apoptosis of Vascular Endothelial Cells. Int. J. Clin. Exp. Pathol. 6 (11), 2376–2385. PubMed PMID: 24228099. 24228099 PMC3816806

[B38] Sun Z. Liu B. Luo Z.-J. (2020). The Immune Privilege of the Intervertebral Disc: Implications for Intervertebral Disc Degeneration Treatment. Int. J. Med. Sci. 17 (5), 685–692. PubMed PMID: 32210719. 10.7150/ijms.42238 32210719 PMC7085207

[B39] Tang Y. Scheef E. A. Wang S. Sorenson C. M. Marcus C. B. Jefcoate C. R. (2009). CYP1B1 Expression Promotes the Proangiogenic Phenotype of Endothelium through Decreased Intracellular Oxidative Stress and Thrombospondin-2 Expression. Blood 113 (3), 744–754. PubMed PMID: 19005183. 10.1182/blood-2008-03-145219 19005183 PMC2628380

[B40] Tang Y. Scheef E. A. Gurel Z. Sorenson C. M. Jefcoate C. R. Sheibani N. (2010). CYP1B1 and Endothelial Nitric Oxide Synthase Combine to Sustain Proangiogenic Functions of Endothelial Cells under Hyperoxic Stress. Am. J. Physiology-Cell Physiol. 298 (3), C665–C678. PubMed PMID: 20032512. 10.1152/ajpcell.00153.2009 PMC2838582 20032512

[B41] Thongnak L. Pongchaidecha A. Lungkaphin A. (2020). Renal Lipid Metabolism and Lipotoxicity in Diabetes. Am. J. Med. Sci. 359 (2), 84–99. PubMed PMID: 32039770. 10.1016/j.amjms.2019.11.004 32039770

[B42] Vernon-Roberts B. Moore R. J. Fraser R. D. (2007). The Natural History of Age-Related Disc Degeneration. Spine (Phila Pa 1976) 32 (25), 2797–2804. PubMed PMID: 18246000. 10.1097/BRS.0b013e31815b64d2 18246000

[B43] Wang J. Lin C. Li H. Li R. Wu Y. Liu H. (2017). Tumor-infiltrating γδT Cells Predict Prognosis and Adjuvant Chemotherapeutic Benefit in Patients with Gastric Cancer. Oncoimmunology 6 (11), e1353858. PubMed PMID: 29147601. 10.1080/2162402X.2017.1353858 29147601 PMC5674957

[B44] Wang Y. Dai G. Li L. Liu L. Jiang L. Li S. (2019). Transcriptome Signatures Reveal Candidate Key Genes in the Whole Blood of Patients with Lumbar Disc Prolapse. Exp. Ther. Med. 18 (6), 4591–4602. PubMed PMID: 31777557. 10.3892/etm.2019.8137 31777557 PMC6862187

[B45] Wang G. Xu J. Zhao J. Yin W. Liu D. Chen W. (2020). Arf1-mediated Lipid Metabolism Sustains Cancer Cells and its Ablation Induces Anti-tumor Immune Responses in Mice. Nat. Commun. 11 (1), 220. PubMed PMID: 31924786. 10.1038/s41467-019-14046-9 31924786 PMC6954189

[B46] Ward J. M. Nikolov N. P. Tschetter J. R. Kopp J. B. Gonzalez F. J. Kimura S. (2004). Progressive Glomerulonephritis and Histiocytic Sarcoma Associated with Macrophage Functional Defects in CYP1B1-Deficient Mice. Toxicol. Pathol. 32 (6), 710–718. PubMed PMID: 15580705. 10.1080/01926230490885706 15580705

[B47] Yasuma T. Makino E. Saito S. Inui M. (1986). Histological Development of Intervertebral Disc Herniation. J. Bone Jt. Surg. 68 (7), 1066–1072. PubMed PMID: 3745246. 10.2106/00004623-198668070-00015 3745246

[B48] Yorimitsu E. Chiba K. Toyama Y. Hirabayashi K. (2001). Long-term Outcomes of Standard Discectomy for Lumbar Disc Herniation: a Follow-Up Study of More Than 10 Years. Spine (Phila Pa 1976) 26 (6), 652–657. PubMed PMID: 11246379. 10.1097/00007632-200103150-00019 11246379

[B49] Zhang B. Horvath S. (2005). A General Framework for Weighted Gene Co-expression Network Analysis. Stat. Appl. Genet. Mol. Biol. 4, Article17. Article17. PubMed PMID: 16646834. 10.2202/1544-6115.1128 16646834

[B50] Zhang X. Chen J. Huang B. Wang J. Shan Z. Liu J. (2019). Obesity Mediates Apoptosis and Extracellular Matrix Metabolic Imbalances via MAPK Pathway Activation in Intervertebral Disk Degeneration. Front. Physiol. 10, 1284. PubMed PMID: 31649558. 10.3389/fphys.2019.01284 31649558 PMC6796795

